# Predictive Factors for and Complications of Bronchiectasis in Common Variable Immunodeficiency Disorders

**DOI:** 10.1007/s10875-022-01206-8

**Published:** 2022-01-11

**Authors:** Johannes M. Sperlich, Bodo Grimbacher, Veronika Soetedjo, Sarita Workman, Siobhan O. Burns, David M. Lowe, John R. Hurst

**Affiliations:** 1 Department of Clinical Immunology, Royal Free London NHS Foundation Trust, London, UK; 2 Center for Chronic Immunodeficiency, Faculty of Medicine and Medical Center, University of Freiburg, Freiburg, Germany; 3 Institute for Medical Biometry and Statistics, Faculty of Medicine and Medical Center, University of Freiburg, Freiburg, Germany; 4 Institute of Immunity and Transplantation, University College London, London, UK; 5 UCL Respiratory, University College London, London, UK

**Keywords:** Common variable immunodeficiency disorders, Bronchiectasis, Forced expiratory volume in one second, Immunoglobulin M, St George Respiratory Questionnaire

## Abstract

Bronchiectasis is a frequent complication of common variable immunodeficiency disorders (CVID). In a cohort of patients with CVID, we sought to identify predictors of bronchiectasis. Secondly, we sought to describe the impact of bronchiectasis on lung function, infection risk, and quality of life. We conducted an observational cohort study of 110 patients with CVID and an available pulmonary computed tomography scan. The prevalence of bronchiectasis was 53%, with most of these patients (54%) having mild disease. Patients with bronchiectasis had lower median serum immunoglobulin (Ig) concentrations, especially long-term IgM (0 vs 0.25 g/l; *p* < 0.01) and pre-treatment IgG (1.3 vs 3.7 g/l; *p* < 0.01). CVID patients with bronchiectasis had worse forced expiratory volume in one second (2.10 vs 2.99 l; *p* < 0.01) and an annual decline in forced expiratory volume in one second of 25 ml/year (vs 8 ml/year in patients without bronchiectasis; *p* = 0.01). Patients with bronchiectasis also reported more annual respiratory tract infections (1.77 vs 1.25 infections/year, *p* = 0.04) and a poorer quality of life (26 vs 14 points in the St George’s Respiratory Questionnaire; *p* = 0.02). Low serum immunoglobulin M concentration identifies patients at risk for bronchiectasis in CVID and may play a role in pathogenesis. Bronchiectasis is relevant because it is associated with frequent respiratory tract infections, poorer lung function, a greater rate of lung function decline, and a lower quality of life.

## Introduction

Common variable immunodeficiency disorders (CVID) are a heterogeneous group of primary immunodeficiencies in which patients fail to produce adequate levels of immunoglobulins [3]. Population-based studies estimate the prevalence of CVID at 14 in 100,000, making it the most common clinically significant primary immunodeficiency [25]. The clinical hallmark of almost all subtypes of CVID is recurrent respiratory tract infections [2]. Many CVID patients also suffer from inflammatory complications, emphasizing the complex interplay between infection and immune dysregulation [9, 12]. Immune dysregulation and immunodeficiency may result in more severe infections and more damaging inflammation. The result of this vicious cycle of infection, inflammation, and structural damage in the lungs is bronchiectasis [6].

Bronchiectasis is a radiological diagnosis based on the demonstration of abnormally dilated bronchi. This is often associated with airway wall thickening, and generally considered to be permanent. Bronchiectasis is the commonest manifestation of chronic lung disease in CVID affecting up to 23% of CVID patients across Europe [7] and may progress despite adequate immunoglobulin replacement [20]. With an increasing prevalence of roughly 5 per 1,000 in the general population, bronchiectasis is not a rare disease and is known to be associated with increased morbidity and mortality [18]. In populations presenting with bronchiectasis, a primary immunodeficiency is found in 4–9% of adult patients [13].

Cough and duration of disease in CVID correlate with the presence of bronchiectasis [23]. Various immunological markers have been proposed to distinguish patients with and without bronchiectasis in CVID: low serum immunoglobulin A and M concentrations at the time of diagnosis, low pre-treatment IgG concentration, and low CD4 count [10, 17, 19, 21] are all factors previously demonstrated to be associated with bronchiectasis in CVID. Exacerbation frequency, lung function parameters, and mortality appear similar in patients with bronchiectasis with or without underlying immunodeficiency [8].

Although we know that immunodeficiencies contribute significantly to bronchiectasis, there is a lack of knowledge about the pathophysiological connection between immune dysfunction and bronchiectasis [28]. In this cross-sectional study, we aimed to investigate associations between on- and pre-treatment immunological markers and bronchiectasis. Secondly, we wanted to explore the impact of bronchiectasis on quality of life, infection risk, and lung function in a cohort of CVID patients. Lung function decline and infection risk inform the clinician about active disease.

## Methods

### Study Design and Study Setting

This study was designed as a retrospective cohort study among CVID patients attending the joint Immunology-Respiratory service at the Royal Free Hospital, London, UK. Participants were eligible if (1) they had a CVID diagnosis made by a clinical immunologist following the International Collaboration in Asthma, Allergy and Immunology definition [3] and (2) a computed tomography scan evaluated by a consultant radiologist. All were prescribed immunoglobulin replacement and scheduled for regular (at least six-monthly) clinical review. As a screening measure for pulmonary pathology, CT scans are routinely scheduled every five years as part of structured clinical care, while pulmonary function testing is planned annually. Measurement of immunoglobulin titres and immune cell phenotyping are also standard care. All participants provided written, informed consent (REC 04/Q0501/119).

### Participants

**Fig. 1:**
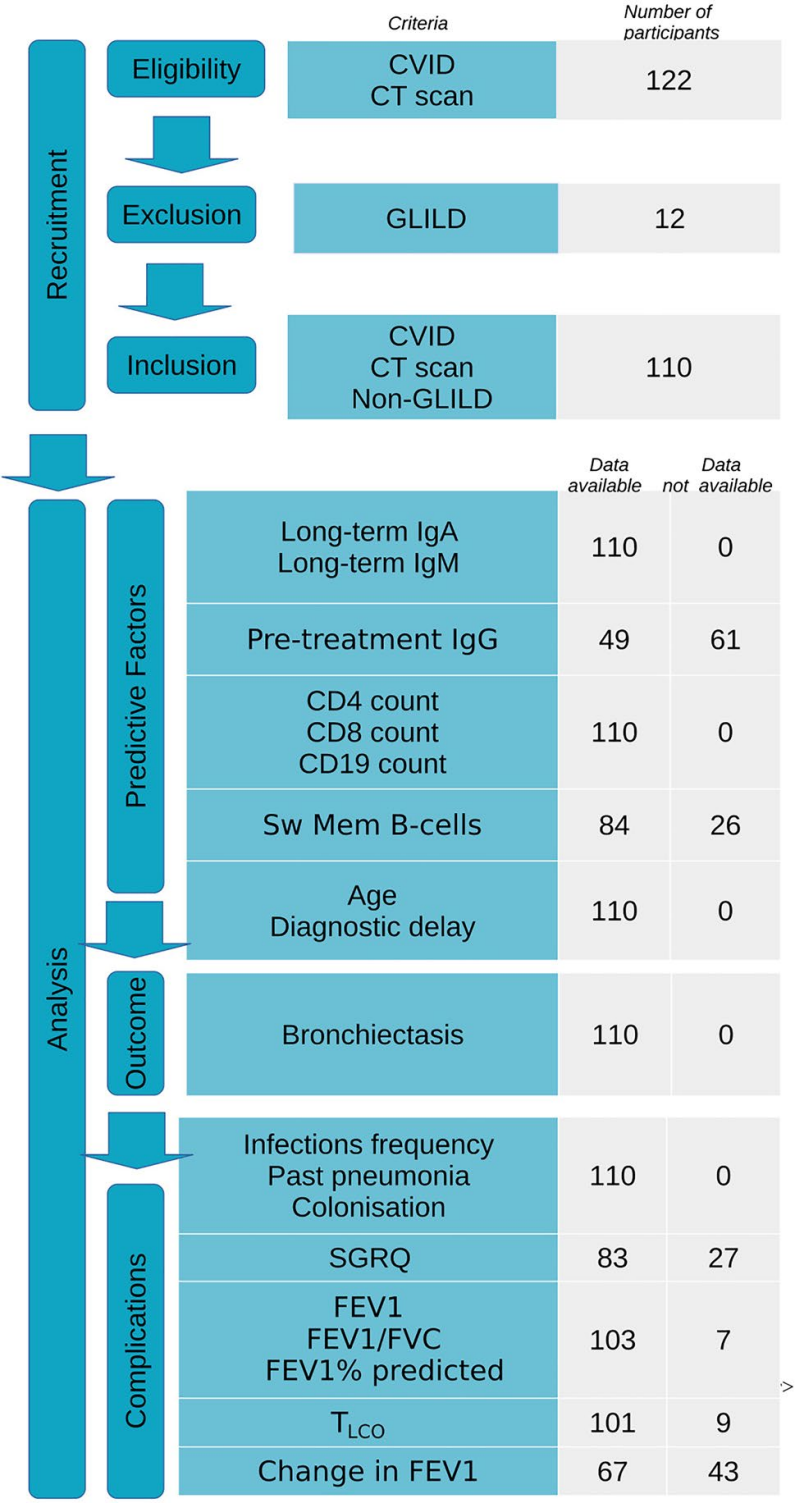
Participants and variable chart. 122 patients with CVID and a CT scan were eligible for our study. We excluded 12 patients with GLILD. Immunological parameters, age and diagnostic delay were independent variables to establish predictors for bronchiectasis. CT scans were assessed for the presence of bronchiectasis (dependent variable). Infection history, quality of life, and lung function were used to compare complications in patients with and without bronchiectasis

One hundred twenty-two CVID patients with an available CT scan were identified and assessed for the presence of radiological abnormalities using the radiologist’s report. Twelve were diagnosed with granulomatous-lymphocytic interstitial lung disease (GLILD). As patients with this subtype of CVID represent a pathology distinct from CVID with infections and bronchiectasis [11], these patients were excluded. Thus, 110 CVID patients with a recent CT scan were included in this study. Figure 1 shows the study design, selection of participants, variables, and data availability.

### Variables and Data Collection

#### Computed Tomography (CT) Scanning

We collected all available radiology reports from CT scans. The scans were evaluated for the presence or absence of bronchiectasis, defined as an airway that is non-tapering, and/or greater in diameter than its accompanying bronchial artery. Patients with only traction bronchiectasis due to pulmonary fibrosis were considered not to have bronchiectasis.

#### Predictive Factors for Bronchiectasis

Medical records of all 110 participants were evaluated for serum IgG concentration at the time of presentation (prior to CVID diagnosis and initiation of immunoglobulin replacement). The follow-up from baseline IgG at diagnosis to the CT scan was a median of six (IQR 4–10) years. Serum IgA and IgM concentrations were measured at least annually at routine clinical visits. To reflect long-term average IgA and IgM serum concentration, we calculated the median values from a total of 1972 measurements over 384 patient years in 110 patients. For each patient, the average immunoglobulin concentrations were calculated from 17 (IQR 11–23) measurements covering four (IQR 3–7) years.

Immune cell phenotyping was performed by flow cytometry in our diagnostic laboratories at the Royal Free Hospital, London. If multiple measurements were available, we used the measurement performed the longest time before the CT scan as a baseline value. We analyzed the following compartments of the T- and B-cell populations: absolute counts of CD4 + , CD8 + , and CD19 + lymphocytes, and proportion of CD27 + IgD- (switched memory cells) within CD19 + B-cells. The follow-up for baseline CD4, CD8, and CD19 counts was two (IQR 0–3) years. For switched memory B cells, the follow-up from baseline to CT scan was one (IQR 0–2) year.

All cell counts and serum immunoglobulin concentrations were standardized prior to analysis. A standardized score is calculated by subtracting the population mean from the raw score and dividing the difference by the standard deviation. Standardization facilitates comparisons of variables with skewed distributions and variables on different scales. To assess additional predictive factors for the development of bronchiectasis, we recorded the patients’ age at the time of the CT scan and the diagnostic delay. Diagnostic delay is defined as the time from onset of symptoms, as reported by the patient, to the start of immunoglobulin replacement therapy.

#### Collection of Clinical Data, Quality of Life, Lung Function, and Bronchiectasis Severity

To describe respiratory health in CVID patients with and without bronchiectasis, we collected clinical data, data on quality of life, and lung function results. Clinical data were collected by means of a standardized questionnaire which participants completed at least once yearly at routine clinical visits. We calculated long-term mean values for the frequency of respiratory infections per year. A respiratory infection was defined by the use of additional antibiotics for a change in respiratory symptoms, as reported by the patient. Airway colonization was diagnosed when the same species of pathogenic bacterium had been isolated more than twice within the two years before our study. Seventy-two patients unable to provide a sputum sample were considered not to be colonized.

We asked patients to complete the St George’s Respiratory Questionnaire (SGRQ), a validated measure of respiratory health-status scoring between 0 and 100 with higher scores indicating poorer health status [14, 30].

All available results from lung function testing were retrieved and we recorded values for the forced expiratory volume in one second (FEV1), FEV1% predicted, FEV1/FVC (forced vital capacity) ratio, T_LCO_ (carbon monoxide transfer factor), and T_LCO_ % predicted. We used results from the lung function test performed closest to the CT scans. In patients with three or more lung function results over more than two years, we assessed annual change in FEV1 calculating the slope of a linear regression line using all available data. We assessed a longitudinal change in FEV1 for 67 patients covering 412 patient years in total.

We used clinical, radiological, and microbiological data to calculate the Bronchiectasis Severity Index (BSI) for patients with bronchiectasis. Data were available for 54 of 58 patients. The BSI assesses the clinical severity of bronchiectasis by differentiating patients with mild, moderate, and severe bronchiectasis and predicts future mortality, hospitalization, and exacerbations [5].

### Statistical Analysis

To compare patients with and without bronchiectasis, we used the Wilcoxon rank sum test for continuous variables and Fisher’s exact test for categorical variables. We used Spearman’s rank correlation to measure correlation between continuous variables. To identify predictive factors for bronchiectasis, we applied simple logistic regression models to individual candidate variables with bronchiectasis as the binary dependent variable. We used all available data in a complete case analysis.

In a second step, we calculated a multiple logistic regression model and accounted for missing variables with multiple imputation. We selected all candidate variables with a *p*-value < 0.1 in simple regression, namely long-term serum IgM concentration, pre-treatment serum IgG concentration, CD4 count, CD19 count and age. In a third step we examined relationships between immunological candidate variables and the presence of bronchiectasis. We performed a principal component analysis to identify groups of inter-related variables. These groups are summarized as “factors” and explain the largest amount of variability in the data. We retained all factors with an eigenvalue of > 1.

Among the predictive factor variables, there were missing data for pre-treatment serum IgG concentration (61/110 missing) and proportion of CD27 + IgD-/CD19 + B-cells (26/110 missing). Missing data were imputed using multiple imputation on the assumption that data were missing at random. This approach is considered superior to a complete case analysis in retrospective analysis to avoid bias and loss of power [27, 29]. The candidate protective factors for bronchiectasis considered in this study and their respective data availability are shown in Fig. 1.

## Results

### Low Serum Concentrations of Non-substituted IgG and IgM Are Associated with Bronchiectasis

**Fig. 2:**
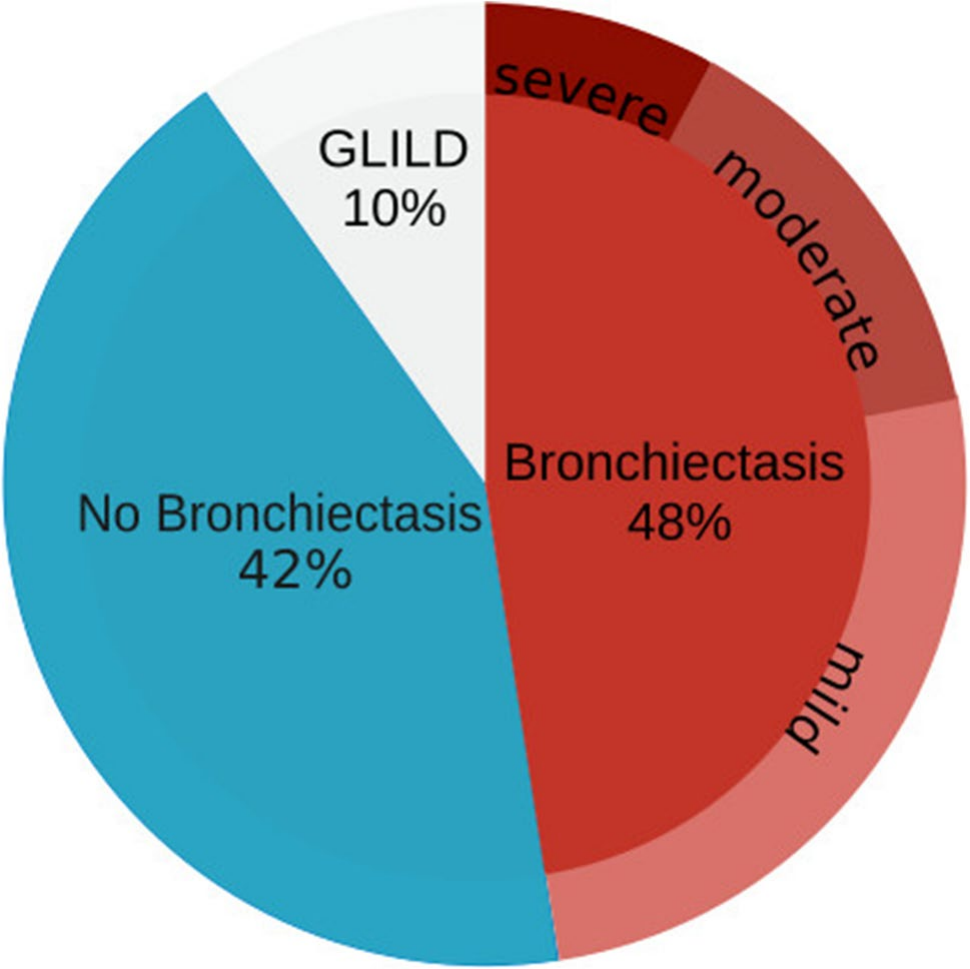
Bronchiectasis prevalence and severity. The frequency of bronchiectasis in a cohort of 122 CVID patients was 48% (58/122). Of patients with bronchiectasis, 54% had clinically mild, 30% moderate, and 16% severe bronchiectasis as defined by the Bronchiectasis Severity (Chalmers 2014). The Bronchiectasis Severity Index predicts future mortality, hospitalization, and exacerbations. Patients with severe bronchiectasis have a 1-year morality rate of 9.9–29.2%, with moderate bronchiectasis of 0.8–4.8%, and with mild 0–2.8% (Chalmers 2014)

CT scans, infection history, lung function results, and respiratory health status were assessed in 110 CVID patients. The data are presented in Table 1. The prevalence of bronchiectasis was 53% (58/110, Fig. 2).

**Table 1:** Patient characteristics

	Total cohort	Bronchiectasis	No bronchiectasis	*p*-value
Number	110 (100)	58 (53)	52 (47)	
Age (years)	50 (38–61)	52 (41–62)	49 (38–55)	0.06
Age at CVID diagnosis (years)	34 (21–46)	35 (21–51	33 (20–46)	0.38
Female	60 (55)	35 (60)	25 (48)	0.14
Diagnostic delay (years)	6 (2–14)	7.5 (2–16)	5 (2–12)	0.11
Frequency of Respiratory Infections per Year	1.46 (0.63–2.65)	1.77 (0.98–2.95)	1.25 (0.45–2.40)	0.04
Known bacterial airway colonization	8 (7)	5 (8)	3 (6)	0.42
Prophylactic antibiotics (% of group)	66 (60)	37 (64)	29 (56)	0.44
Macrolides (% of group)	39 (35)	20 (34)	19 (36)	0.84
Βeta-lactams (% of group)	10 (9)	7 (12)	3 (6)	0.34
SGRQ total score*	22 (8–40)	26 (15–46)	14 (5–38)	0.02
FEV1 (l)	2.52 (1.92–3.34)	2.10 (1.78–2.89)	2.99 (2.32–3.65)	0.0003
Annual change in FEV1 (ml/year)	− 18 (− 39 to − 4)	− 25 (− 43 to − 10)	− 8 (− 29–12)	0.01
FEV1 predicted (%)	93 (75–104)	84 (68–99)	99 (87–108)	0.002
FEV1/FVC ratio (%)	75 (68–80)	73 (66–78)	77 (70–81)	0.03
T_LCO_ (mmol/min/kPa)	6.98 (5.38–8.97)	6.27 (4.66–7.88)	7.76 (5.96–9.50)	0.004
T_LCO_ predicted (%)	81 (67–92)	76 (63–84)	86 (72–98)	0.009
Long-term average serum IgG trough concentration (g/l)†	8.7 (7.6–9.7)	8.8 (7.7–10.0)	8.6 (7.3–9.6)	0.35
Long-term average serum IgM concentration (g/l)	0.1 (0.0–0.3)	0 (0.0–0.1)	0.25 (0.05–0.7)	0.0001
Long-term average serum IgA concentration (g/l)	0 (0–0)	0 (0–0)	0 (0–0.2)	0.61
Pre-treatment serum IgG concentration (g/l)§	2 (0.9–4.1)	1.3 (0.5–2.5)	3.7 (1.5–4.4)	0.006
Baseline CD4 + T-cell count per µl	630 (444–857)	498 (410–732)	747 (568–917)	0.03
Baseline CD8 + T-cell count per µl)	407 (274–621)	422 (274–658)	392 (276–581)	0.13
Baseline CD19 + B-cell count per µl	173 (73–278)	142 (48–257)	188 (107–340)	0.07
Baseline Sw mem B-cells %**	1.56 (0.06–4.39)	0.78 (0.00–2.67)	2.02 (0.64–5.92)	0.11

Data are median (IQR) or *n* (%)

^*^SGRQ, St George’s Respiratory Questionnaire, a validated measure of respiratory health-status scored between 0 (best) and 100 (worst) quality of life

^†^, serum IgG trough concentration, serum immunoglobulin G level measured immediately before an immunoglobulin infusion

^§^, pre-treatment serum IgG concentration, serum immunoglobulin G level measured before immunoglobulin replacement was started

^**^Sw mem B-cells, proportion of CD27 + IgD-/CD19 + B-cells

*p*-values for all predictor variables were taken from simple logistic regression models. All other *p*-values were calculated using to a Wilcoxon rank sum test for continuous variables and Fisher's exact test for categorical variables

**Fig. 3:**
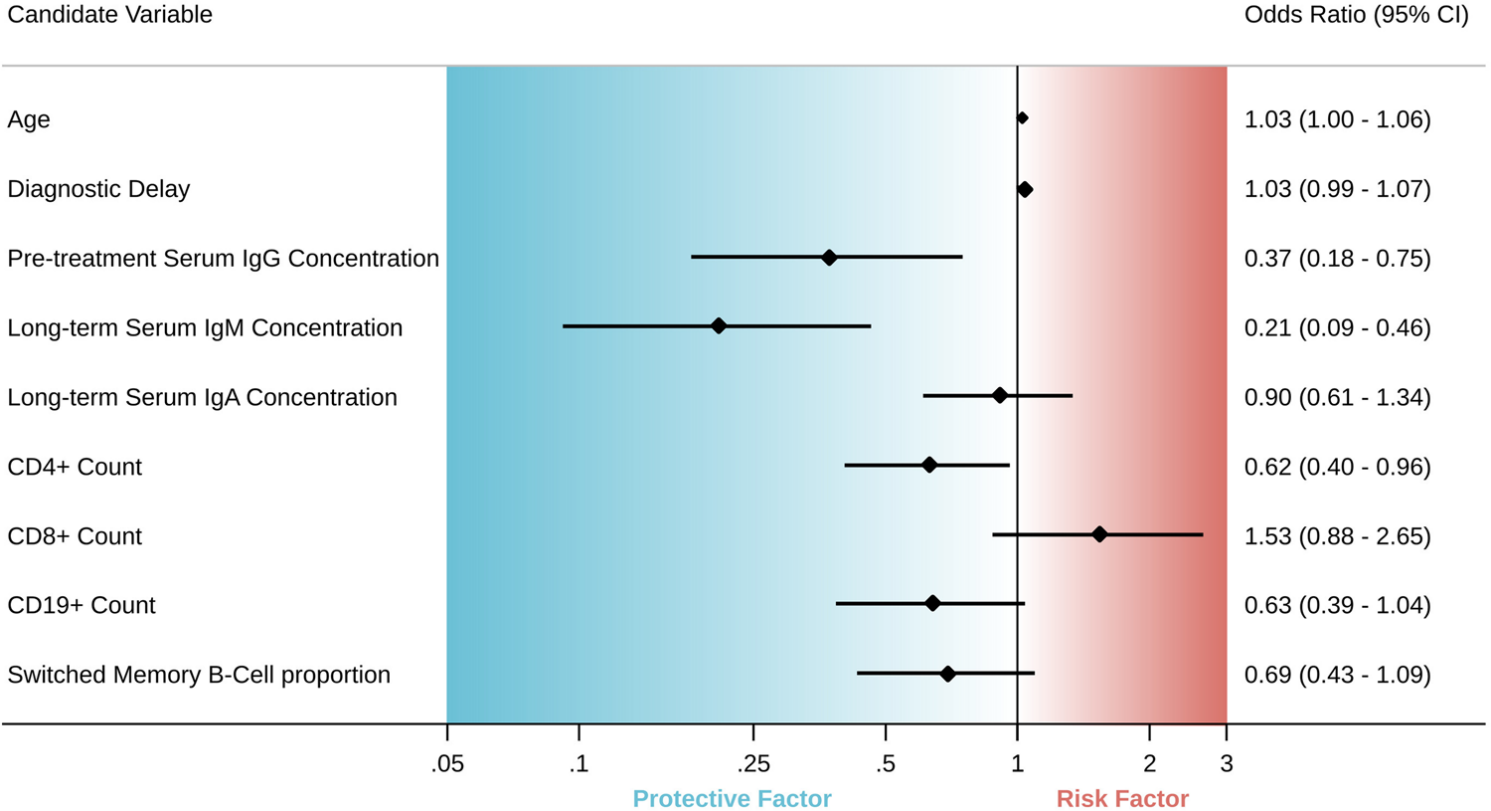
Associations with bronchiectasis in simple logistic regression. The plot displays odds ratios and 95%-CI as a measure of association between candidate variables and bronchiectasis. Odds ratios below one suggest that higher concentrations of the variable are associated with a lower risk for bronchiectasis (protective factor), while odds ratios above one suggest that higher concentration of the variable is associated with a higher risk for bronchiectasis (risk factor)

Figure 3 shows the results from simple logistic regression of serum immunoglobulin concentrations and lymphocyte counts on the presence of bronchiectasis. Of all tested predictors, we found that higher serum IgM concentration was associated with the greatest reduction in risk of bronchiectasis (OR = 0.20; 95%-CI = 0.09–0.46). This indicates that for each increase of 0.18 g/l in serum IgM concentration, the odds of bronchiectasis halve.

High pre-treatment serum IgG concentrations were also associated with a reduced risk of bronchiectasis (OR = 0.37; 95%-CI = 0.18–0.75). This indicates that for each increase of 1.30 g/l pre-treatment IgG the odds of bronchiectasis halve. There was no statistically significant association between low serum IgA concentration and bronchiectasis (OR = 0.90; 95%-CI = 0.61–1.34).

Higher CD4 count was associated with a decreased risk of bronchiectasis (OR = 0.62; 95%-CI = 0.40–0.96). CD8 and C19 counts, and CD27 + IgD-/CD19 + B-cells were not associated with the presence of bronchiectasis in simple logistic regression. There was no significant association between diagnostic delay or age and bronchiectasis.

In order to identify independent predictive factors for bronchiectasis, we calculated a multiple regression model. When adjusting for other variables, low serum IgM concentration remained the only independent, significant predictor of bronchiectasis (OR = 0.24; 95%-CI = 0.10–0.59). There was no correlation between bronchiectasis and any other variable.

### Immunoglobulins and Lymphocyte Counts Are Each Inter-related

As our immunological predictors may be inter-related, we sought to identify underlying factors which accounted for the greatest variation. We conducted a principal component factor analysis which identified two independent factors dominated by immunoglobulin concentration and lymphocyte counts respectively.

**Fig. 4:**
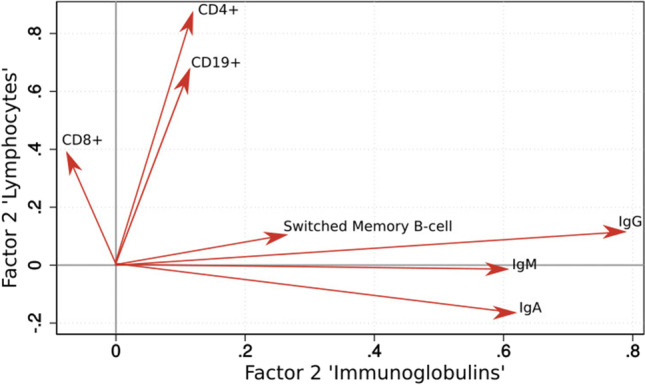
Factor loadings. The loading plot shows the association of variables with the two factors which we identified in the principal component analysis. Long-term serum IgM and IgA concentration and pre-treatment IgG concentration were positively correlated; they contribute to the factor “Immunoglobulins”. CD4, CD8, and CD19 counts were correlated; they contributed to the factor “Lymphocytes.” The proportion of switched memory B-cells had little weight on either of the other two factors

Factor loadings were highest for lymphocytes (CD4 + , CD8 + , CD19 +) in Factor 1 and for immunoglobulins (IgM, IgA, pre-treatment IgG) in Factor 2. IgM, IgA, and pre-treatment IgG contributed to the same factor and were positively correlated. Likewise, CD4, CD8, and CD19 also contributed to one factor and were positively correlated. The proportion of switched memory B-cells had factor loadings < 0.3 and was therefore considered not to be associated with either of the other two factors. Figure 4 shows the association of variables with the two factors.

In multiple logistic regression, we found Factor 2 “Immunoglobulins” to be associated with bronchiectasis (OR = 0.18; 95%-CI = 0.04–0.70), indicating that low concentrations of immunoglobulin A, M, and pre-treatment IgG were associated with bronchiectasis. Factor 1 “Lymphocytes” was not independently associated with bronchiectasis.

### Bronchiectasis in CVID Is Associated with More Rapid Decline in Lung Function, a Higher Rate of Respiratory Infections, and Lower Quality of Life

Fifty-three percent (58/110) of patients had bronchiectasis. Most (54%) patients with bronchiectasis had mild bronchiectasis as classified by the Bronchiectasis Severity Index; 30% had moderate and 17% had severe bronchiectasis (Fig. 2). Patients with bronchiectasis had a lower quality of life as measured by the St. George’s Respiratory Questionnaire. Patients with bronchiectasis had a higher total SGRQ score compared to patients without bronchiectasis (median (IQR) 25.94 (14.82–45.60) vs 14.20 (4.54–38.33); *p* = 0.02). The Bronchiectasis Severity Index correlated with the SGRQ (*ρ* = 0.79; *p* < 0.001) indicating that more severe bronchiectasis is associated with a lower quality of life. FEV1 and the SGRQ also were correlated (*ρ* =  − 0.38; *p* < 0.001) indicating that a lower FEV1 is associated with a lower quality of life.

There was greater expiratory air flow limitation in patients with bronchiectasis compared to those without. FEV1 was lower in patients with bronchiectasis (median FEV1 (IQR) 2.10 (1.78–2.89)l vs 2.99 (2.32–3.65)l; *p* < 0.001). Accordingly, FEV1% predicted was also lower in patients with bronchiectasis although still within normal limits (median FEV1 predicted (IQR) 84 (68–99)% vs 99 (87–108)%; *p* = 0.002). FEV1/FVC was lower in patients with bronchiectasis (median FEV1/FVC (IQR) 73 (66–78)% vs 77 (70–81); *p* = 0.03). Diffusion capacity was also lower in patients with bronchiectasis (median T_LCO_ predicted (IQR) 76 (63–84)% vs 86 (72–98)%; *p* = 0.009).

**Fig. 5:**
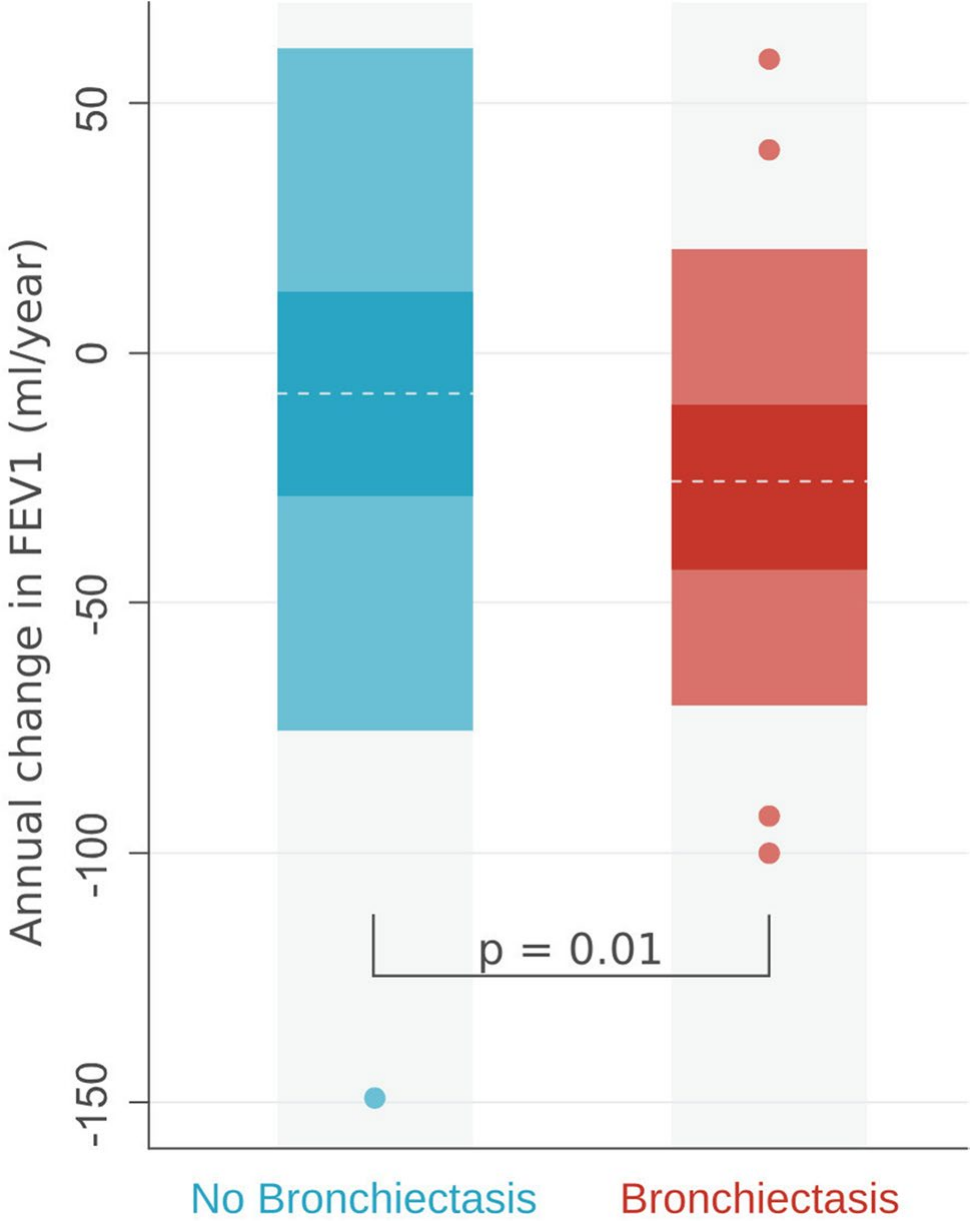
Annual change in FEV1 in 67 patients with and without bronchiectasis. Patients with bronchiectasis have significantly higher median FEV1 decline than patients without (− 25 vs − 8 ml/year, *p* = 0.01). The boxes extend from lower to upper quartile with a line at the median. Whiskers extend to lower and upper adjacent value

Multiple lung function results were available for 67 patients in whom we calculated change in FEV1 over time. The overall median (IQR) change in FEV1 was − 18.25 (− 39.25 to − 3.95)ml/year. Annual decline in FEV1 was greater in patients with bronchiectasis than in patients without (median dFEV1/dt (IQR) − 25.35 (− 43.66 to − 10.50) ml/year vs − 8.02 (− 28.70–12.16) ml/y; *p* = 0.01). Figure 5 compares annual change in FEV1 in patients with and without bronchiectasis.

Patients with bronchiectasis reported a greater number of annual respiratory tract infections than patients without bronchiectasis (median (IQR) 1.77 (0.98–2.95) infections/year vs 1.22 (0.43–2.45) infections/year; *p* = 0.04). A lower serum IgM concentration was associated with a higher number of annual respiratory tract infectious (*ρ* =  − 0.23; *p* = 0.02).

Eight (7%) patients were colonized with pathogenic bacteria. There was no significant difference in the prevalence of colonization in patients with or without bronchiectasis, although numbers with demonstrable colonization were low. However, in those with bacterial colonization, bronchiectasis was clinically more severe (BSI 2 vs 3; *p* = 0.03), and serum IgM concentration was lower (0.0 vs 0.1 g/l; *p* = 0.02). Sixty-six (60%) patients were on prophylactic antibiotics, but colonized patients were not more likely to be on prophylactic antibiotics.

## Discussion

In this observational study of 110 patients with CVID, excluding those with concomitant GLILD, the prevalence of bronchiectasis was 53%, with most of these patients (54%) having mild disease. Patients with bronchiectasis had lower serum immunoglobulin concentrations, especially long-term IgM and pre-treatment IgG. CVID patients with bronchiectasis had poorer lung function and higher annual FEV1 decline. Patients with bronchiectasis also reported more annual respiratory tract infections and a lower quality of life as measured by the SGRQ.

Low pre-treatment IgG concentration, low IgA concentration, and low CD4 + T cell counts are factors known to be associated with bronchiectasis in CVID [4, 17, 19]. Our study supports these findings. However, in contrast to these studies, we found low serum IgM concentration to be the strongest predictor of bronchiectasis. Immunoglobulin concentrations are co-correlated and we demonstrated an association between overall immunoglobulin levels and bronchiectasis. We thus hypothesize that high serum immunoglobulin concentrations, particularly of non-substituted IgG and IgM, protect against bronchiectasis.

Pre-treatment IgG levels and long-term IgM levels likely distinguish CVID patients with more severely impaired B-cell function from CVID patients with residual B-cell function and this serves as a risk factor for the development of bronchiectasis. We hypothesize that patients with detectable levels of IgM might be more able to clear pathogens via T cell–independent IgM antibody production whereas patients with undetectable levels of IgM cannot clear pathogens as rapidly and thus develop bronchiectasis.

While IgG is replaced in patients with CVID, IgM is currently not. The results of administering intravenous IgM-enriched immunoglobulin G in sepsis are still debated [1, 15]. Our study suggests that higher long-term average IgM protects against bronchiectasis and raises the question as to whether IgM substitution may prevent the development or positively influence the course of bronchiectasis in immunodeficiency.

CVID patients without bronchiectasis are known to have better lung function in a cross-sectional comparison [26]. In our longitudinal study, we have shown that FEV1 decline in CVID patients without bronchiectasis is only eight ml/year. As a comparison, a decline of up to 30 ml/year is considered typical in healthy non-smokers [16] but there is much individual variability in lung function decline. CVID patients without bronchiectasis also reported fewer respiratory tract infections. This indicates lower pulmonary disease activity in CVID patients without bronchiectasis. We have previously shown that CVID-associated bronchiectasis presents a distinct inflammatory phenotype [22].

The impact of bronchiectasis on the quality of life of patients with CVID is profound. With a minimal clinically important difference for the SGRQ score of four units [14], the difference of twelve units between patients with and without bronchiectasis here indicates a highly clinically relevant effect. This emphasizes the importance of identifying patients at risk of bronchiectasis (for example those with low pre-treatment IgG and low long term IgM) and optimizing their care, including educating patients about the importance of promptly treating respiratory infections. This is important as we have previously shown that patients with CVID tend to delay and avoid treatment of symptomatic respiratory exacerbations, which could result in structural lung damage [24]. We have previously shown that the quality of life impact in CVID relates most closely to respiratory involvement, including the severity of airflow obstruction and respiratory exacerbation frequency [12].

Our study has some limitations. We retrieved data on the presence of bronchiectasis using radiological reports from computed tomography scans. While we assessed clinical severity using the BSI, we did not independently assess the radiological severity of bronchiectasis. Using an observational study design, we can only present associations between bronchiectasis and other variables. Although biologically plausible, we cannot assume cause and effect in the development of bronchiectasis. Trying to maximize the number of eligible participants, our dataset was not complete for all variables in all subjects. We accounted for missing variables by complete case analysis and multiple imputation. As cases and controls were recruited from the same cohort, a systematic bias is unlikely. However, CVID is a phenotypically and genetically heterogeneous disease. Missing unknown confounders remain a possibility. We collected sputum samples only when patients were able to spontaneously produce sputum during clinical visits. Colonized patients unable to spontaneously produce sputum may have gone unnoticed.

In conclusion, this study suggests that CVID patients with bronchiectasis have impaired lung health as measured by low FEV1, low T_LCO_, high SGRQ, more respiratory infections per year, and a higher annual rate of FEV1 decline. This is not true and in fact is reversed in those CVID patients without bronchiectasis. This study has demonstrated the profound impact of bronchiectasis on patients with CVID, making better identification of those at risk an important objective. This can be facilitated using standard laboratory markers — particularly pre-treatment IgG and on-treatment IgM.

## Data Availability

The data can be shared on reasonable request to the corresponding author if the privacy of individuals that participated in the study allows it.

## Abbreviations

BSI: Bronchiectasis Severity Index [5]
CT: Computed tomography scan
CVID: Common variable immunodeficiency disorders
FEV1: Forced expiratory volume in one second
FEV1/FVC: Proportion of forced expiratory volume in one second to forced vital capacity
FEV1% predicted: FEV1 expressed as a % predicted for a person of that age, sex, height and race
IgA: Immunoglobulin A
IgG: Immunoglobulin G
IgM: Immunoglobulin M
IQR: Interquartile range
OR: Odds ratio
SGRQ: St George’s Respiratory Questionnaire [14]
T_LCO_: Transfer factor of the lung for carbon monoxide
T_LCO_ predicted: Predicted T_LCO_ expressed as a % predicted for a person of that age, sex, height, and race

## Code Availability

Statistical analysis was performed using Stata Version 14.0 (StataCorp LP, College Station, TX, USA). The code can be shared on reasonable request to the corresponding author.
